# Transmission of stimulus-induced epigenetic changes through cell division is coupled to continuous transcription factor activity

**DOI:** 10.3389/fimmu.2023.1129577

**Published:** 2023-03-14

**Authors:** Sarah Sun, Raúl Aguirre-Gamboa, Luis B. Barreiro

**Affiliations:** ^1^ Committee on Immunology, University of Chicago, Chicago, IL, United States; ^2^ Medical Scientist Training Program, The University of Chicago, Chicago, IL, United States; ^3^ Department of Human Genetics, University of Chicago, Chicago, IL, United States; ^4^ Section of Genetic Medicine, Department of Medicine, University of Chicago, Chicago, IL, United States

**Keywords:** innate immune memory, macrophages, histone modifications, immunogenomics, epigenetics, trained immunity

## Abstract

Trained immunity, or innate immune memory, has been attributed to the long-term retention of stimulus-induced histone post-translational modifications (PTMs) following clearance of the initial stimulus. Yet, it remains unknown how this epigenetic memory can persist for months in dividing cells given the lack of any known mechanism for stimulus-induced histone PTMs to be directly copied from parent to daughter strand during DNA replication. Here, using time course RNA-seq, ChIP-seq, and infection assays, we find that trained macrophages are transcriptionally, epigenetically, and functionally re-programmed for at least 14 cell divisions after stimulus washout. However, the epigenetic changes observed after multiple rounds of cell division do not result from the self-sustained propagation of stimulus-induced epigenetic changes through cell division. Instead, long-lasting epigenetic differences between trained and non-trained cells are always coupled with changes in transcription factor (TF) activity, emphasizing the central role played by TFs, and gene expression changes more broadly, in driving the transmission of stimulus-induced epigenetic changes across cell divisions.

## 1 Introduction

Immunological memory has historically been considered an exclusive hallmark of the adaptive immune response. In recent years, however, emerging evidence has shown that prototypical innate immune cells can also mount resistance to reinfection, a phenomenon termed “trained immunity” (1, 2). Although trained immunity has emerged as a focal point in immunology research there are still many unknowns regarding the mechanisms required for its development and maintenance. Memory in innate immune cells has primarily been linked to histone post translational modifications (PTMs) (3–7). Histone modifications occur at well-defined regulatory regions such as enhancers and promoters (8–10) and are highly dynamic in response to stimulation (5, 11, 12). *In vitro*, monocyte-derived macrophages stimulated with B-glucan (BG) gain histone modifications H3K4me1, H3K4me3, and H3K27Ac at regulatory regions and these changes can be detected throughout their *in vitro* lifespan (3, 6, 7). These macrophages are reprogrammed (“trained”) to secrete higher levels of proinflammatory cytokines IL6 and TNFA upon a secondary challenge (3, 6, 7). Due to the correlative nature of these studies, however, we do not know the extent to which changes in histone modifications are causally required for the induction of trained macrophages.

In dividing cells, such as tissue resident macrophages or pluripotent stem cells, the situation is further complicated. During DNA replication parental PTMs are distributed onto only one of two daughter strands and synthesized *de novo* on the other. Thus, if the newly synthetized histones are not modified post-replication, the information encoded in histone PTMs will be rapidly lost by dilution in successive rounds of cell division. Moreover, frequent histone exchange post-replication, which is expected to primarily occur in active chromatin regions, can further challenge the inheritance of histone PTMs (13). Supporting that view, a recent study found preservation across the cell cycle of biotinylated histones in repressed domains, but not among transcriptionally active sites (14, 15). This suggests that histone PTMs that bookmark active regions – thought to be central for the trained immunity phenotype (6) – should be short-lived, which is hard to reconcile with the long-term effects – sometimes in the scale of several years post primary stimulus – described for trained immunity-like states (16–20). To date, most of the studies that have investigated long-term effects of trained immunity in self-renewing cell types have focused on tissue resident macrophages (21, 22) or hematopoietic stem and progenitor cells (HSPCs) using mouse models (17, 23, 24). While these approaches may be the best way to study trained immunity in the biological context they pose barriers to answering basic questions related to the inherent ability of dividing cells to retain epigenetic signatures without input from other cell types in the microenvironment or from continued low-level stimulus-persistence.

Here we explored the mechanistic basis of innate immune memory in an isolated, dividing macrophage population through dense time course transcriptional, epigenetic, and functional profiling of macrophages following stimulation and washout of BG – a common trained immunity inducing stimulus. We find that trained macrophages are transcriptionally, epigenetically, and functionally re-programmed for at least 14 cell divisions after stimulus washout. However, epigenetic signatures remaining through multiple rounds of cell division are coupled with the continued differential activity of transcription factors, suggesting that they may not be self-sustained. We find that many of the differences observed at late timepoints arise not from retention of BG-induced signatures, but from new waves of coupled transcription factor activity and H3K4me1 deposition beginning days following stimulus washout. Thus, our data points to a dynamic process, as opposed to static retention and propagation of histone modifications, as underlying long-lasting trained immunity within isolated, dividing macrophages.

## 2 Material and methods

### 2.1 Cells and reagents

LM1 cells were a gift from Dr. Martin Olivier (McGill University) and were cultured in DMEM supplemented with 10% heat-inactivated FBS and pen-strep glutamine unless indicated otherwise in T75 flasks (Falcon). NFκB GFP reporter LM1 cells were cultured in DMEM supplemented with 10% heat-inactivated FBS and pen-strep glutamine and 2.5 ug/mL puromycin (Sigma) unless indicated otherwise in T75 flasks. DMEM supplemented with 10% heat inactivated FBS and L-glutamine (Gibco) was used during all steps of *Salmonella Typhimurium* infections.

Beta glucan was provided by D. Williams (East Tennessee State University) and was added to a final concentration of 30 ug/mL in all experiments.

Bone marrow derived macrophages were generated to verify that the LM1 response to BG resembles that of primary macrophages. Bone marrow derived macrophages from the bone marrows of C57BL/6 mice were generated as previously described (17) but using DMEM (Gibco) supplemented with 10% heat-inactivated FBS (Gibco) and penicillin-streptomycin-glutamine (Gibco) in place of RPMI and 25 ng/mL M-CSF (Prospec protein specialists) instead of 30% L929-conditioned media.

All mice used for BMDM generation were housed in the University of Chicago animal facility in accordance with the policies of, and approved by, the University of Chicago Institutional Animal Care and Use Committee. Mice were housed under SPF conditions.

### 2.2 Monoclonal reporter generation

LM1 cells were transduced with lentiviral particles (Qiagen) containing multiple repeats of the NFκB promoter driving the expression of GFP, using SureENTRY (Qiagen) at an MOI of 10. After 24 hours cells were washed with PBS and given new media. To isolate stably transduced clones, we added 2.5 ug/mL puromycin (Sigma) to the cell culture medium for 24 hours and allowed the cells to reach confluency (~5 days). Cells were harvested and single cells were sorted into 96-well plates. After 2 weeks, reporter activity of individual clones was tested by stimulating clones with beta glucan followed by flow cytometry analysis of GFP fluorescence. One highly responsive clone was chosen and used for all subsequent experiments.

### 2.3 Flow cytometry analysis of reporter activity

To assess NFκB activity at various timepoints following beta glucan washout or in direct response to stimulation, cells were harvested by removing media, washing with PBS, and incubating adherent cells in 10 mL Accutase (Sigma) for 5 mins at room temp. Detached cells were pooled, washed 1x with PBS, and resuspended in 1% BSA in PBS. Cells were analyzed immediately on a Fortessa flow cytometer (BD Biosciences) using the FITC channel to measure GFP fluorescence. Data was analyzed using FlowJo version 10.

### 2.4 EdU labelling

To estimate LM1 cell division rates, EdU incorporation time course assays were performed using Click-iT™ Plus EdU flow cytometry assay kit (Invitrogen) per manufacturer’s instructions. Briefly, naïve LM1 cells or LM1 cells stimulated with 30 ug/mL beta glucan 24 hours prior were incubated with 10 µM EdU for 0, 6, 9, 12, 13, 14, or 15 hours as indicated. After the incubation, cells were harvested, washed once with 1% BSA in PBS and fixed in 100 μL of Click-iT™ fixative for 15 minutes at room temperature. Following fixation, cells were washed with 1% BSA in PBS and incubated for 15 minutes in 100 μL of 1X Click-iT™ permeabilization and wash reagent. Cells were stained with Click-iT™ Plus reaction cocktail containing fluorescent Pacific Blue picolyl azide, incubated for 30 minutes at room temp in the dark, washed with 1X Click-iT™ permeabilization and wash reagent, and analyzed on a Fortessa (BD biosciences).

In initial optimizations to estimate LM1 cell divisions we tried using CellTrace, however LM1 cells appeared to divide as a highly homogenous population, meaning that at any given time, after staining for CellTrace and analyzing the cells using Flow Cytometry, we saw only a single peak shifting across the x-axis (with decreasing mean fluorescent intensity) over time, rather than seeing discrete peaks showing clear populations of cells undergoing variable numbers of cell division.

### 2.5 Cell sorting and post-sort culture of time course experiments

#### 2.5.1 Cell collection for sorting

Prior to sorting, reporter LM1 macrophages (either unstimulated or stimulated with beta glucan for 24 hours), were harvested by removing all media, washing with PBS, and incubating adherent cells in Accutase at room temp for 5-10 mins. Detached cells were pooled, washed with PBS, and resuspended in PBS to a concentration of approximately 1 million cells per milliliter. Cells were filtered through a 40 µM sterile filter. Live-dead stain was performed for all samples by addition of 1:10 of a 10 µg/mL propidium iodide solution 10 minutes prior to sorting. Cells were sorted on a FACSAria II or FACSAria IIIu instrument. For controls, we sorted all PI^neg^ singlets. For BG-stimulated cells, we sorted all PI^neg^, singlet, GFP^+^ cell.

*Post-sort cell culture:* Sorted control and BG-experienced cells were washed with PBS, resuspended in media, counted, and seeded into 2 T75 flasks each (~2M for next day D2 collection and 0.6M cells for harvest/passage at D4). Only 0.6M cells were seeded into the D4 flask to prevent cells from reaching confluency prior to harvest. Cells were collected at D2, D4, …, up to D14 for ChIP-seq, RNA-seq, and secondary stimulation. At D4 harvest, remaining cells not collected for ChIP-seq, RNA-seq, and secondary stimulation were seeded into 2 new T75 flasks (repeating the same process again, 2M for next day D6 collection and 0.6M cells for harvest at D8). The same process of harvest and collection was repeated up to D14.

#### 2.5.2 Cell harvest

At each harvest timepoint, cells were harvested using Accutase to detach cells, followed by a wash with 1x PBS. Cells were aliquoted into tubes for downstream ChIP-seq or RNA-seq processing. Whenever possible, we aimed to collect 3M cells for ChIP-seq and at least 0.5M cells for RNA-seq. A subset of cells was plated into four wells of a 6-well plate (600,000 cells per well) and stimulated with Pam3CSK4 to assess priming (conditions: control unstim, BG unstim, control + pam, BG + pam). After 5 hours of stimulation, plated cells were harvested in Accutase for RNA extraction and sequencing.

### 2.6 RNA extractions

Harvested cells were washed with PBS and lysed with 1mL of Qiazol. RNA extractions were performed using the miRNeasy mini or miRNeasy micro kits (Qiagen). RNA quality was evaluated with the 2100 Bioanalyzer (Agilent Technologies).

### 2.7 RNA sequencing

RNA library preparations were carried out on 100-500 ng of RNA with RIN > 9 using the Illumina TruSeq Stranded Total RNA Sample preparation kit, according to the manufacturer’s instructions. The libraries were size-selected using Ampure XP Beads (Beckman Coulter) and quantified using the KAPA Library Quantification kit - Universal (KAPA Biosystems). Sequencing of the RNA-Seq libraries was performed on the Illumina NovaSeq 6000 system using 100-bp single-end sequencing. Gene expression levels across genes were strongly correlated across all pairs of samples (R>0.975) attesting for the high quality of the dataset.

### 2.8 ChIP-sequencing

Samples collected at various timepoints of the post-BG time course were crosslinked with 1% v/v formaldehyde for 10 mins shaking at 50 rpm at room temperature and then quenched for 5 minutes by addition of 1.25M Glycine to a final concentration of 125 mM. Formaldehyde fixed samples were sonicated for 12 mins using a Biorupter (Diagenode) with 105 peak power and 200 cycles/burst. Sonicated chromatin was split into equal parts for each targeted immunoprecipitation. ChIP-DNA was prepared by incubating chromatin with antibody (anti-H3K4me1, anti-H3K4me3, or anti-H3K27Ac) for at least 10 hours at 4C on a thermomixer set to 300 rpm, followed by Dynabeads (protein A) incubation for 2 hours and washes with Low Salt wash buffer, High Salt wash buffer, LiCl wash buffer, and TE buffer. The number of input cells used for each ChIP ranged from 0.5 million to 1 million. The following antibodies were used: H3K4me1 (company: CST; ref: 5326S), H3K4me3 (company: CST; ref: 9751S), H3K27Ac (company: Abcam; ref: ab4729). ChIP and input libraries were prepared using the diagenode MicroPlex Library Preparation kit (v3) and sequenced on the Illumina NovaSeq 6000 system at the University of Chicago Genomics Facility using 100-bp paired-end sequencing. A subset of samples was sequenced a second time to obtain greater sequencing depth across all samples and histone modifications. These samples were sequenced 100-bp single-end on the Illumina NovaSeq 6000 system at the University of Chicago Genomics Facility.

### 2.9 Salmonella Typhimurium infections

#### 2.9.1 Salmonella culture

Salmonella Typhimurium was grown in TSB media (15g TSB in 500 mL DI water). For infections, the concentration of bacteria was determined by taking the OD_600_ and diluting bacteria in media to a concentration of 1.5M bacteria per milliliter of solution.

#### 2.9.2 Bacterial infections

Control and beta-glucan experienced LM1 macrophages were infected with *Salmonella Typhimurium* at various time points following beta glucan washout to compare functional ability. Approximately 12 hours prior to the desired time of infection, *Salmonella Typhimurium* cultures were established by incubating a small aliquot of frozen bacterial stock in TSB media at 37C and rotations set to 250 rpm. On the day of infections, cells were seeded at a density of 300,000 cells per well in 6-well plates containing 2 mL media 2 hours prior to infection. Cells were infected for 40 minutes with Salmonella Typhimurium at an MOI=10 by removing supernatant from attached cells, washing with pre-warmed Phosphate-buffered saline, and adding 2 mL per well of bacterial solution (see ‘Salmonella culture’ above). After 40 minutes, bacterial solution was removed, and cells were washed with pre-warmed PBS. Media containing 50 µg/mL gentamycin was added to cells which were then incubated at 37C for 1 hour to kill any remaining extracellular bacteria. Following 1-hour incubation (designated Time0/T0), cells were washed and new media containing 3 µg/mL gentamycin was added to cells.

### 2.10 Colony forming unit assay

To quantify the kinetics of intracellular bacterial growth *in vitro*, cells were harvested at 2- and 6-hours post-infection (T2 and T6 respectively) by removing all supernatant and washing cells with PBS. Cells were lysed to release intracellular bacteria by adding 1 mL sterile water supplemented with 1% TritonX-100 and pipetting up and down vigorously 10 times. Serial dilutions made in PBS were plated on TSB plates. Plates were incubated at 37C and counted the next day. Bacterial growth was quantified as the fold-change difference in CFUs between the 6-hour and 2-hour plates.

### 2.11 RNA-seq data processing

Adaptor sequences and low-quality score bases were first trimmed using Trimmomatic with parameters -phred33 SE ILLUMINACLIP : TruSeq3-SE.fa:2:30:10 LEADING:3 TRAILING:3 SLIDINGWINDOW:4:15 MINLEN:36 (25). The resulting reads were aligned to the mm10 mouse reference genome using STAR (26). Read counts are obtained using featureCounts (27) with default parameters.

### 2.12 Differential gene expression analyses

For each RNA-sequencing data set, gene expression levels across all samples were first normalized using the calcNormFactors function implemented in the edgeR R package (version 3.34.0) which utilizes the TMM algorithm (weighted trimmed mean of M-values) to compute normalization factors. Then, the voom function implemented in the limma package (version 3.38.3) was used to log-transform the data and to calculate precision-weights. A weighted fit using the voom-calculated weights was performed with the lmFit function from limma.

#### 2.12.1 Effect of BG stimulation on gene expression

To investigate the impact of beta-glucan stimulation on LM1 cells at various timepoints of stimulation (2, 4, 7, 12, 15, and 18 hours), normalized, log-transformed gene expression levels were fit to the linear model Expression ~ 1 + time + stimulus:time, which corrects for natural changes across time independent of beta-glucan stimulation and therefore captures the independent effect of beta-glucan on gene expression at each time point (BG at 2 hrs., BG at 4 hrs., …, BG at 18 hours). LM1 cells stimulated for 24 hours, and their respective controls were collected in an additional set of extra experiments. Normalized gene expression for these samples was fit to the linear model Expression ~ 1 + experiment + stimulus to correct for “batch” effects between replicate samples while giving an independent estimation of the impact of 24-hours BG stimulation on gene expression.

#### 2.12.2 Effect of previous BG exposure on expression

For samples collected post-BG washout in the time course experiment, the design Experiment ~1 + experiment + time + primary:time + ((Pam:time):primary) was used to correct for batch effects between replicate experiments and natural variations across time while giving an estimate of the lasting impact of previous BG exposure on gene expression at each time point (effect of BG on expression at D2, D4, etc.).

#### 2.12.3 Hierarchical clustering

Normalized, log-transformed expression values were also used for downstream hierarchical clustering analysis. Expression values were corrected to remove the effect of experimental variation and natural across-time changes by subtracting the “experiment” and “time” effects from all samples. Corrected, normalized expression values were scaled and centered such that each gene had a mean = 0 and sd = 1 across all samples. Scaled expression was used to compute a distance matrix between samples using the dist function in R with parameters: method = “Euclidean” and diag=TRUE. Samples were clustered based on calculated Euclidean distances using the function hclust and viewed as a dendrogram using the fviz_dend function implemented in the R package factoextra (version 1.0.7) with parameters: k=7, type=phylogenetic.

#### 2.12.4 Effect of pervious BG exposure on secondary responses to Pam3CSK4

To investigate whether BG-experienced LM1 cells were primed at various time points post-washout, we used the design Expression ~1 + experiment + primary + Pam:primary to estimate priming (the effect of Pam secondary stimulation on naïve/control cells compared to the effect of Pam on BG-experienced cells) independently for each time point using the makeContrasts and contrasts.fit functions implemented in limma. To increase our power to detect primed genes shared or unique to each time point we applied Multivariate Adaptive Shrinkage (28) in R (mashr version 0.2.57) to outputs from contrasts.fit. Effect sizes were obtained from contrasts.fit in limma and the standard error of the effect size for each gene was given by multiplying the square root of the posterior variance by the standard deviation for effect size. Effect sizes and standard errors for each time point (n = 6; D2, D4, D6, D10, D12, D14) were arranged into n x m matrices, n being the number of genes and m being the number of timepoints. We then fit the mash model using canonical and data driven covariance matrices and then stringently defined primed genes as those with an lfsr < 0.01.

### 2.13 Gene ontology analysis

We used the enrichGO function implemented in the clusterProfiler R package (version 4.0.5) to identify gene ontology terms enriched among DE genes (FDR<0.05, abs(logFC)>1) in LM1 cells at 7 hours of beta glucan stimulation. We used the parameters: OrgDb = “org.Mm.eg.db”, ont = “BP”, pAdjustMethod = “BH”, minGSSize = 10, maxGSSize = 500. The same parameters were used to determine gene ontology enrichments among genes grouped into specific trajectories (increasing, constant, and decreasing).

### 2.14 GSEA

Gene set enrichment analyses (GSEA) were performed using the fgsea R package (version 1.18.0) with parameters: maxSize = 500, nperm=100000. To investigate biological pathway enrichments among DE genes during BG stimulation, or at various timepoints after BG washout, genes at each time point were ordered by the rank statistic: -log10(pvalue)*logFC and compared with the Hallmark gene sets from the MSigDB collections.

Enrichments for primed genes after Pam secondary stimulation were performed using the same parameters. For each time point, genes were ordered by the mashr calculated values: -log10(lfsr)*PosteriorMean and compared with the Hallmark gene sets from the MSigDB collections.

### 2.15 ChIP-seq data processing

ChIP-seq reads were mapped to the mouse reference genome (GRCm38/mm10) using Bowtie2 with default parameters. Mapped reads were sorted and filtered using the samtools functions *view* (with parameter -q 30 to retain only high-quality mapped reads), and *sort* with default parameters. PCR duplicates were removed using Picard MarkDuplicates program with parameter “REMOVE_DUPLICATES=True”. Peaks were subsequently called independently for each sample using the callpeak function from the MACS2 software suite (29) with parameters -q 0.05 –keep-dup all. For each histone modification, a merged peak file combining called peaks across all samples was generated using the bedops *merge* function with default parameters. Read counts overlapping the merged peak set were obtained using featureCounts (27).

### 2.16 ChIP-seq analysis/differential testing

For downstream ChIP-seq analysis, samples containing fewer than 7 million aligned reads were removed from the raw count matrix. Initial processing of the filtered ChIP-seq count data was performed as described for RNA-seq data: The number of reads overlapping each peak across all samples for a given histone modification were normalized using the calcNormFactors function implemented in the edgeR R package (version 3.34.0). Then, the voom function implemented in the limma package (version 3.38.3) was used to log-transform the data and to calculate precision-weights. A weighted fit using the voom-calculated weights was performed with the lmFit function from limma.

#### 2.16.1 Effect of previous BG exposure on histone modification levels

For samples collected post-BG washout in the time course experiment, the design Experiment ~1 + experiment + time + primary:time was used to correct for batch effects between replicate experiments and natural variations across time while giving an estimate of the lasting impact of previous BG exposure at each time point (effect of BG on histone PTM levels at D2, D4, etc.).

### 2.17 ChromHMM

We used ChromHMM (30, 31) to segment the genome into gene regulatory states for control (n=3 replicates) and BG-experienced cells (n=3 replicates) at D14 using aligned histone PTM bam files for H3K4me1, H3K4me3, and H3K27Ac. Briefly, we first used BinarizeBam with default parameters (binsize=200) to learn chromatin states in 200 bp intervals for each histone PTM. Then we used the LearnModel function (default parameters except n=7 emission states) to partition the genome into states based on the combinatorial presence or absence of each histone PTM. We manually assigned each emission state to a gene regulatory state as follows: H3K4me1^lo^/H3K4me3^hi^/H3K27Ac^hi^ – “promoter_active”, H3K4me1^hi^/H3K4me3^hi^/H3K27Ac^hi^ – “enh/prom_active”, H3K4me1^hi^/H3K4me3^lo^/H3K27Ac^hi^ – “enhancer_active”, H3K4me1^lo^/H3K4me3^lo^/H3K27Ac^lo^ – “none”, H3K4me1^hi^/H3K4me3^lo^/H3K27Ac^lo^ – “enhancer_inactive”, H3K4me1^hi^/H3K4me3^hi^/H3K27Ac^lo^ – “enh/prom_inactive”.

### 2.18 Motif enrichment analysis

Motif enrichment analyses were performed using the Homer (32) function findMotifsGenome with parameters -size 1000 -mask. For motif enrichment analyses performed on subsets of H3K4me1 peaks (i.e., “increasing” or “not-increasing”), we used the entire set of called H3K4me1 peaks (n=122,962) as background.

### 2.19 Gene networks of post-washout increasing genes

We used the interferome database (interferome.org) containing manually curated sets of type I, II, and III interferon genes to predict which genes characterized as “increasing” during the post-BG washout period were predicted to be regulated by IRF7 or STAT1. We defined each increasing gene as belonging to the same network as IRF7, STAT1, or both based on the presence or absence of the respective TF motifs in the gene’s promoter region. Network visualization was performed using cytoscape (v3.8.2) in which all circles represent genes in the increasing trajectory, and lines connecting genes to IRF7, STAT1, or both indicate a likely binding of the respective transcription factors to the gene’s promoter region.

### 2.20 Relationship between significant H3K4me1 peaks and genes of different trajectories

To investigate the genomic association between H3K4me1 peaks and genes following the same trajectories, we assigned H3K4me1 peaks following “induced”, “retained”, or “non-persistent” trajectories to their nearest genes using the Homer function *annotatePeaks* with default parameters. Background peaks were defined as all H3K4me1 peaks and foreground peaks were defined as peaks in the “induced”, “retained”, or “non-persistent” trajectories. Enrichment for genes within matching trajectories was performed using a two-sided Fisher’s exact test in R.

## 3 Results

### 3.1 iBMDM^NFκB-GFP^ cells enable dense time course profiling of epigenetic and gene transcriptional dynamics after BG stimulation

**Figure 1:**
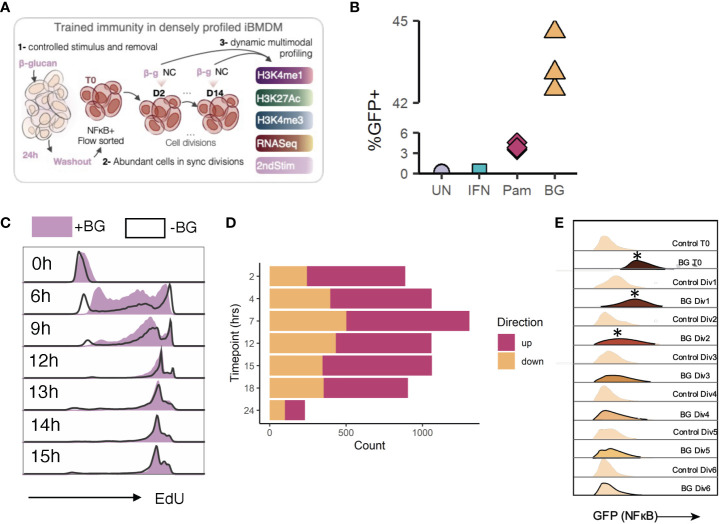
Experimental model design and validation. **(A)** General experimental plan. **(B)** Quantification of percent GFP^+^ cells among unstimulated cells, or cells stimulated with IFN (negative control), Pam3CSK4, or BG. Data are representative of 3 replicates per condition. **(C)** Naïve (no stimulus) iBMDM^NFκB-GFP^ cells or iBMDM^NFκB-GFP^ cells previously stimulated with 30 µg/mL beta glucan were incubated with EdU for 6, 9, 12, 13, 14, or 15 hours. At each time point, paired unstimulated and post-stimulation cells were collected. EdU was fluorescently labelled (BV421) by Click-it reaction. EdU incorporation was quantified by flow cytometry (gated on single cells). **(D)** Quantification of the number of differentially expressed genes (Log2FC>1, FDR<0.05) in iBMDMs after various hours of BG stimulation. **(E)** Histograms from flow cytometry analysis of GFP levels (FITC channel) in BG^exp^ cells over time relative to paired controls.

We sought to investigate how BG exposure affects the transcriptional, epigenetic, and functional profile of dividing macrophages over the course of many cell divisions following stimulus removal. Specifically, we wanted to choose an experimental setup that would allow 1) controlled stimulation and subsequent stimulus removal, 2) synchronous cell divisions, and 3) a sufficient population of pure cells to enable multimodal profiling at many timepoints (**Figure 1A**). Although we explored the possibility of using cultured primary bone marrow derived macrophages (BMDMs) as a pure population of dividing macrophages, we found it difficult to control their division rates and to passage these cells for an extended period of time. This prompted us to explore the possibility of using immortalized BMDMs (iBMDMs) as a representative, and easily manipulatable, model of primary BMDMs.

We first generated a reporter iBMDM line to enable a fluorescent readout of cell activation at any point during or after stimulation. We transduced iBMDMs (mouse BMDMs immortalized by infection with J2 retrovirus (33)) with lentiviral particles containing an NFκB inducible GFP construct and then clonally selected successfully transduced iBMDMs to generate monoclonal reporter cells (hereafter referred to as iBMDM^NFκB-GFP^), which express GFP when NFκB is active. A population of GFP+ cells emerged after stimulation of iBMDM^NFκB-GFP^ cells with BG or Pam3CSK4 (a known NFκB inducer), but not IFNα, confirming the specificity of the reporter and its ability to distinguish activated from non-activated cells within a population (**Figure 1B**). To characterize the division rates of our reporter line, we incubated iBMDM^NFκB-GFP^ cells with EdU (a thymidine analog that is incorporated into actively replicating DNA), harvested cells at multiple timepoints, and fluorescently labelled EdU such that the extent of incorporation could be detected using flow cytometry. Using this method, we found that EdU positivity first reached a maximum in cells incubated for 12 hours, implying full replication of DNA across all cells within this time frame (**Figure 1C**). EdU positivity also peaked at 12 hours in the same time course incubation experiment performed on iBMDM^NFκB-GFP^ cells that had previously been stimulated with BG, indicating that, on a population level, both naïve and post-stimulation iBMDM cells undergo one cycle of DNA replication within approximately 12 hours.

Having established a reporter system with defined division rates, we characterized the initial gene expression response of iBMDM^NFκB-GFP^ cells to BG stimulation. iBMDM^NFκB-GFP^ cells were stimulated with BG and harvested for RNA-sequencing at 7 timepoints during stimulation to examine genome-wide dynamic changes in gene expression. iBMDM^NFκB-GFP^ cells rapidly upregulated hundreds of immune genes (**Figures 1D**, **S1A**; **Table S1**). The number of responsive genes peaked after 7 hours of BG stimulation, although 232 genes (0.05% FDR, abs(Log2FC)>0.5) remained at 24 hours (**Figure 1D**). The response of iBMDM^NFκB-GFP^ cells to BG was comparable to the one engaged by primary BMDMs (**Figure S1B**; Pearson’s r = 0.61, P< 1x10^-16^, 82% concordant in the direction of the effects), supporting their validity as an experimental model to study gene regulatory responses to immune stimulation. To confirm that BG could be fully removed after stimulation, we took advantage of the fact that iBMDM^NFκB-GFP^ cells act as a reporters of cell activation (NFκB activity). We quantified GFP levels among sorted iBMDM^NFκB-GFP^ GFP+ cells compared to paired control cells at 12-hour intervals (every cell division) following BG washout (**Figure 1E**). GFP levels in the BG-stimulated cells were significantly higher compared to controls immediately following stimulation (T0) and for the next two cell divisions but returned to baseline levels thereafter (**Figures 1E**, **S1C**). Thus, we defined any timepoint beyond 2 cell divisions as representative of an inactivated state. Collectively these data demonstrated that iBMDM^NFκB-GFP^ cells divide within 12 hours, induce an immune response to BG resembling that of primary BMDMs, become strongly and selectively GFP+ in response to BG activation, and return to an inactivated state within 3 cell divisions after BG washout.

### 3.2 BG-experienced cells have long-lasting H3K4me1 signatures of previous BG exposure

To assess the long-term impact of BG on iBMDM^NFκB-GFP^ cells, and to investigate whether stimulus-induced histone modifications could be propagated though cell divisions independently of continued NFκB activation, we designed a time course experiment to probe gene expression (*via* RNA-seq) and histone PTM levels (*via* ChIP-seq) of histone marks associated with promoters (H3K4 trimethylation, or H3K4me3), enhancers (H3K4 monomethylation, or H3K4me1), and their activation levels (H3K27 acetylation, or H3K27ac) every 2 cell divisions (up to 14 divisions) after washout of a 24-hour BG stimulation. We stimulated iBMDM^NFκB-GFP^ cells with BG for 24 hours and sorted GFP+ cells to enrich for cells that were responsive to the stimulation (**Figures S2A-C**). Following sorting, we returned control (C) and BG stimulated, GFP+ cells (BG^GFP+^) to cell culture (hereafter referred to as BG-experienced/BG^exp^) and collected an aliquot of C and BG^exp^ cells at timepoints corresponding to 2, 4, 6, 8, 10, 12, and 14 cell divisions (referred to as D2, D4, etc.) post sorting for transcriptional and epigenetic profiling (**Figures S2D**, **2A**). Time 0 (T0; immediately after 24 hours BG) samples were collected in a separate set of experiments.

**Figure 2:**
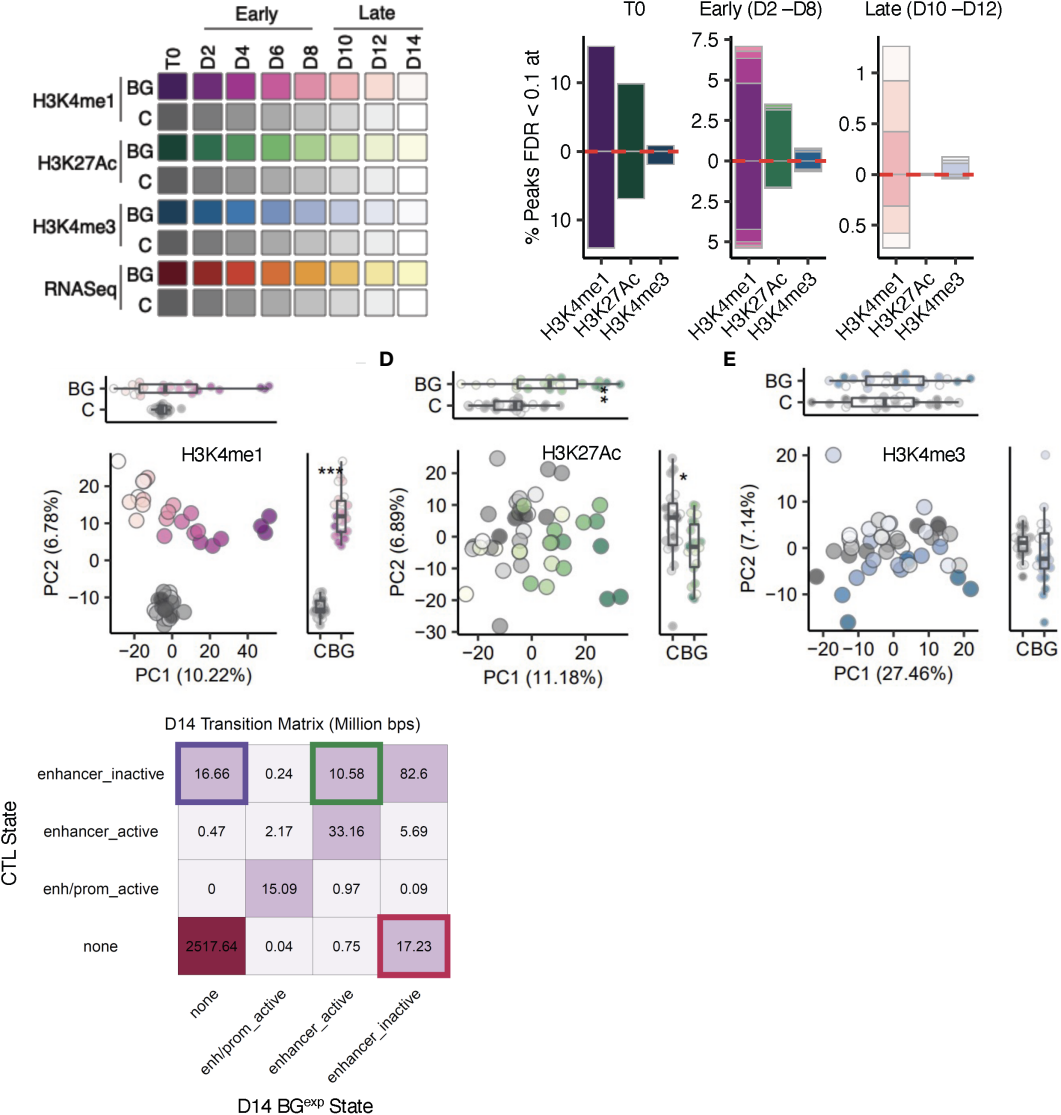
The epigenetic landscape following BG stimulation and washout is characterized by changes in H3K4me1 at enhancer-like regions. **(A)** Sample overview. iBMDM^NFκB-GFP^ cells were left unstimulated (C, gray scale), or stimulated with 30 µg/mL β-glucan (BG, purple, green, blue and red color scales) for 24 hours. Sorted C and BG^GFP+^ cells were returned to cell culture. Aliquots of C and BG^exp^ cells were collected every 24 hours up to 168 hours (D2-D14) and used for downstream RNA-seq and ChIP-seq analysis. **(B)** Bar plots showing the proportion of sites with significantly altered levels of H3K4me1, H3K4me3, and H3K27Ac peaks at T0, early (D2, D4, D6, and D8) and late (D10, D12, and D14) timepoints (colors match the time points indicated in A). Red dotted line marks the direction of effect. **(C)** Principal component analysis (PCA) of H3K4me1 levels in BG^exp^ cells collected between D2 and D14 and time-paired controls across (across 3 experimental replicates). PCs were calculated using log2(counts per million) reads for each sample across all H3K4me1 peaks. **(D, E)**, represent same analysis as in C but for H3K27Ac and H3K4me3. **(F)** Transition matrix displaying the proportion of base pairs in each transition state at D14 related to enhancers (full transition matrix is in **Figure S2G**). ChromHMM was used to segment the genome into 6 states using H3K4me1, H3K4me3, and H3K27Ac ChIP-seq data. Separate segmentations were performed with control and BG D14 ChIP-seq profiles. State assignments between control and BG D14 segmentation outputs were compared across the entire genome using 200 base pairs as the minimal unit.

Using these time course data sets (3 independent replicates per time point), we first explored the impact of BG on genome-wide dynamics of H3K4me3, H3K4me1, and H3K27Ac. We focused on these three histone modifications because of their hypothesized role in encoding innate immune memory (2). For each time point and histone modification, we first quantified the number of sites with significantly (false discovery rate (FDR) < 0.1) altered levels in BG^exp^ compared to paired control samples (**Figures 2B**, **S3A**; **Tables S2-5**). Consistent with evidence that enhancer-associated H3K4me1 and H3K27Ac are more highly responsive to environmental perturbations compared to H3K4me3 (5, 8, 12, 34), we detected larger numbers of sites with altered levels of H3K4me1 and H3K27Ac in BG^exp^ cells collected at T0 immediately after washout (23,679 and 7756 respectively; FDR<0.1, **Figure 2B** *T0*). In total, we detected only 541 peaks (<3% of total peaks tested) with significantly altered levels of H3K4me3 at T0. This number dropped more than 10-fold by D4 and to near-zero levels by D10 (**Figure 2B** *early* and *late* respectively). Likewise, while more than 7000 (>16%) peaks had altered levels of H3K27Ac at T0, this number dropped to only 70 peaks by D4 and to less than 5 peaks as early as D8. H3K4me1 was the most responsive histone PTM, with more than 23,000 significant sites (~29%) at T0 but also declined rapidly – to only ~6,500 sites (~9%) within 2 cell divisions. For all three histone PTMs, the percentage of significant sites decayed exponentially across time, suggesting a lack of mechanisms to preserve these histone PTMs through each round of cell division. Indeed, we found no significant differences in the percentage of histone modifications significant at T0 and remaining significant at each subsequent timepoint as compared to a model of 50% loss with each cell division (**Figure S3B**; K-S test P_H3K4me1_ = 0.9639, P_H3K4me3_ = 0.27, P_H3K27Ac_ = 0.6272), demonstrating that most BG induced histone modifications are readily lost with each successive round of DNA replication.

Despite this being the global pattern, we did detect more than 300 peaks with significantly altered levels of H3K4me1 across the entire time course, even after 14 cell divisions following BG removal (**Figure 2B** *late*). Moreover, principal component analyses (PCA) on H3K4me1 levels revealed a clear and significant separation along PC2 between control and BG^exp^ samples at every timepoint (**Figure 2C**) suggesting an overall rewiring of the H3K4me1 landscape of macrophages upon BG treatment. In contrast, PCA on the levels of H3K4me3 and H3K27Ac revealed no clear separation between control and BG^exp^ samples along either PC1 or PC2, especially at later time points (**Figures 2C-E**).

To further investigate whether BG stimulation induced long-lasting chromatin state transitions (e.g., a transition from an inactive enhancer to an active enhancer), we input H3K4me1, H3K27Ac, and H3K4me3 data sets collected at D14 into ChromHMM (30, 31), which uses a multivariate hidden Markov model to define chromatin states along the genome. Using ChromHMM, we generated genome-wide state segmentations separately for control cells and BG^exp^ at D14 and then determined all regions across the genome for which state assignments differed between C and BG^exp^ (**Figure S3C**). While most of the genome was in the same state in both C and BG^exp^ samples by D14, we detected state transitions at genomic regions covering more than 44M total base pairs. Among enhancer regions, ChromHMM identified the existence of 3 possible enhancer substates: *‘enhancer inactive’* referring to regions that are H3K4me1^hi^H3K27Ac^lo^H3K4me3^lo^, *‘none’* referring to regions that are H3K4me1^lo^H3K27Ac^lo^H3K4me3^lo^ and therefore contain no clear preexisting signatures of enhancers, and ‘enhancer active’ regions which are H3K4me1^hi^H3K27Ac^hi^H3K4me3^lo^. All of the most prevalent state transitions at D14 represented switches between these three enhancer substates. Primarily we identified 3 types of transitions: a complete loss of enhancer activity (‘enhancer_inactive’ to ‘none’), a gain of new enhancer regions *de novo* (‘none’ to ‘enhancer_inactive’), or a switch from an inactive to an active enhancer (‘enhancer_inactive’ to ‘enhancer_active’) (**Figure 2F**). These data show that BG stimulation leads to a long-lasting selective rewiring of the enhancer landscape, which includes both the loss of enhancer activity and the uncovering of novel latent enhancers (5).

### 3.3 Residual H3K4me1 differences are accompanied by changes in gene expression

**Figure 3:**
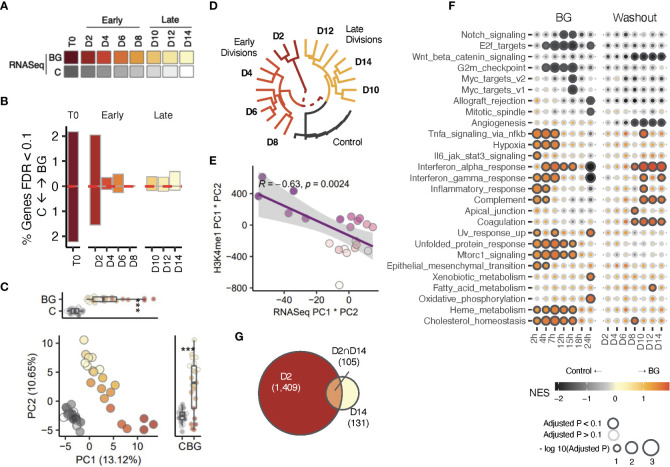
Transcriptional changes correlate with H3K4me1 changes. **(A)** Sample overview for RNA-seq data. iBMDM^NFκB-GFP^ cells were left unstimulated (C, gray scale), or stimulated with 30 µg/mL β-glucan (red color scale) for 24 hours. Sorted C and BG^GFP+^ cells were returned to cell culture. Aliquots of C and BG^exp^ cells were collected every 24 hours up to 168 hours (D2-D14) and used for downstream RNA-seq (here) and ChIP-seq analysis (see **Figure 2**). **(B)** Bar plots showing the proportion of differentially expressed genes (DEG) at T0, early (D2, D4, D6, and D8) and later (D10, D12, and D14). Red dotted line marks the direction of effect. **(C)** Principal component analysis (PCA) of gene expression BG^exp^ cells collected between D2 and D14 and time-paired controls across (across 3 experimental replicates). PCs were calculated using log2(counts per million) reads for each sample across. **(D)** Unsupervised hierarchical clustering analysis of samples based on transformed log2(counts per million) values of the set of differentially expressed genes (FDR<0.05) detected across all time points. Log (CPM) values of control replicates within each time point were averaged and displayed as a single point. **(E)** Correlation analysis of PC1×PC2 values in the PCA using gene expression data (x-axis) compared to PC1×PC2 values in the PCA from **Figure 2C** using H3K4me1 peak values. **(F)** Gene set enrichment analysis of Hallmark pathways performed using the fgsea R package. A separate gene set enrichment analysis was performed at each time point. Genes were ordered in descending order according to -log10(pvalue)×log2FC values (NES is Normalized Enrichment Score). Outlined circles indicate significant (padj<0.1) enrichment and color shading corresponds to NES score as indicated in the legend. Only pathways with significant enrichments for at least one time point are displayed. **(G)** Euler diagram displaying overlapping DEGs between the earliest division (D2) and latest one (D14). * indicates p-value Wilcoxon signed rank test < 0.05, *** indicates p < 0.0005.

At first glance, H3K4me1 profiles suggested that BG^exp^ cells may intrinsically retain a subset of stimulus induced H3K4me1 across multiple cell divisions, even after loss of NFκB activity at D3 (**Figure 1E**). To better understand whether these differences in H3K4me1 were occurring in the presence or absence of any concomitant differential gene expression at all, we used time series RNA-sequencing to quantify the number of significantly differentially expressed (DE) genes between control and BG^exp^ cells (FDR<0.1) at each time point (**Figures 3A, B**; **Table S6**). Not surprisingly, we detected the greatest number of DE genes at T0 and the first BG-washout timepoint, D2. This number dropped precipitously between divisions 2 and 4 from 1578 to 198 DE genes. However, we continued to detect similar numbers of DE genes (>100) at almost all subsequent time points including D14 **(** **Figure 3B**), even when using a stricter p-adjusted cutoff of 0.05 **(** **Figure S3D**). Indeed, PCA revealed that BG^exp^ samples remained clearly distinguishable from all control cells regardless of the collection time point (**Figure 3C**). Unsupervised hierarchical clustering confirmed both the long-lasting separability from control samples, and time point specific clustering of BG^exp^ samples (**Figure 3D**) based on gene expression, suggesting that although BG^exp^ cells rapidly lose NFκB activation, BG exposure may permanently alter the baseline transcriptional state of macrophages. Interestingly, we found that pc1*pc2 values in the gene expression PCA (**Figure 3C**) correlated strongly with pc1*pc2 values of the H3K4me1 PCA (**Figure 2C**), suggesting a relationship between patterns of continued differential gene expression and H3K4me1 levels (**Figure 3E**; correlation coef = -0.626). To better understand the nature of DE genes, we performed a gene set enrichment analysis (GSEA) to look for enriched hallmark pathways at each time point (D2-D14) as well as at multiple time points during BG stimulation (using data shown in **Figure 1D**). As expected, immune-related pathways such as ‘Inflammatory response’ and ‘Tnfa signaling *via* nfkb’, were among the most strongly enriched pathways in direct response to BG stimulation (2-7 hours of stimulation) (**Figure 3F**). Surprisingly, we noticed that most of these BG responsive pathways were no longer enriched early on during the washout period (D2). However, multiple pathways, such as the complement pathway and the interferon alpha/gamma response pathways, reappeared at later timepoints (D10-D14) during the washout period. Other pathways such as Coagulation became enriched only post-BG while never being enriched in direct response to BG. Moreover, when looking at the overlap of DE genes at the first and last timepoints, we found that more than 50% of DE genes at D14 were not significant at D2 (**Figure 3G**), suggesting that continued differential gene expression following BG washout may not be indicative of *retained* immune activation per se, but rather indicative of new waves of expression emerging during the washout period. We considered the possibility that some of these new or reappearing gene expression programs may be the result of post-BG cell apoptosis, however we saw no positive significant enrichment of the Hallmark apoptotic signature at any timepoint, suggesting that these new waves of expression were more likely driven by a cell intrinsic process.

### 3.4 Post-BG changes in the H3K4me1 landscape are coupled to signatures of altered TF activity

Our gene expression and ChIP data collectively suggested that BG^exp^ cells co-regulate their enhancer and gene expression landscapes. We hypothesized that although we could detect differences in H3K4me1 as far out as 14 cell divisions after BG washout, this may be reliant on differential transcription factor activity. If this were the case, we reasoned that the epigenetic changes observed should be intertwined with the remodeling of specific gene expression programs. Specifically, changes in gene expression should be reflective of the activity of the same TFs predicted to bind at sites of altered H3K4me1.

**Figure 4:**
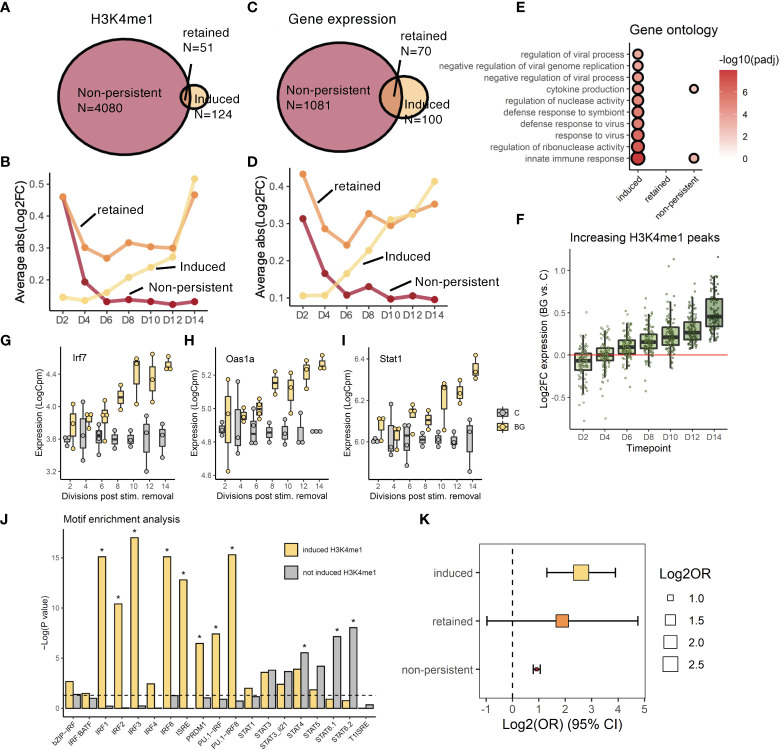
Post-BG changes in the H3K4me1 landscape are coupled to signatures of altered TF activity. **(A)** Venn diagram of showing how H3K4me1 peaks were grouped into 3 trajectories based on differential levels at D2 (non-persistent), D14 (induced), or both timepoints (retained). **(B)** Trajectories of significant (fdr<0.05) H3K4me1 peaks categorized as non-persistent, induced, or retained. Y-axis is the mean absolute value of log2FC differential abundance across all peaks in the group. **(C)** Venn diagram showing how genes were grouped into 3 trajectories based on differential expression at D2 (non-persistent), D14 (induced), or both timepoints (retained). **(D)** Trajectories of significant (fdr<0.05) genes categorized as non-persistent, induced, or retained. Y-axis is the mean absolute value of log2FC differential expression across all peaks in the group. **(E)** Gene ontology analysis (Biological process pathways) on genes within each trajectory. Shown pathways are the top 10 most significantly enriched (fdr<0.05) pathways in the “induced” group. Circle size and color are scaled to –log10(fdr). **(F)** H3K4me1 peaks in the induced trajectory. **(G-I)** Examples of induced genes, Irf7, Oas1a, and Stat1. **(J)** Summary of homer motif enrichment results for interferon motifs performed on induced and non-induced H3K4me1 peaks (* denotes fdr < 0.05). **(K)** Log2 odds ratio (x axis) enrichment of genes in the ‘induced’, ‘retained’, or ‘non-persistent’ trajectories, among genes annotated to peaks in the ‘induced’, ‘retained’, or ‘non-persistent’ trajectories respectively.

To investigate this question, we started by categorizing H3K4me1 peaks and genes into different groups based on their time course trajectories and looking at the relationship between genes and peaks in matched trajectories. For H3K4me1 peaks we focused on those peaks which were significantly differentially abundant (DA, FDR<0.05) at either only the beginning (DA_D2_, N=4080), the end (DA_D14_, N=124), or both timepoints of the time course (DA_D2, D14_, N=51), representing non-persistent, induced, or retained peaks, respectively (**Figures 4A**, **S4A, B**). For each group, we plotted the average absolute log2FC of peaks in the group at each timepoint (**Figure 4B**). The log2FC values of non-persistent peaks declined steadily throughout the washout period while values for induced peaks rose beginning as early as 4 cell divisions after washout. Log2FC values for the small set of retained peaks dipped between D2 and D4 but remained at around 0.3 for most of the washout period before rising back to levels similar to D2 at the end. Using the same approach to split genes in matching trajectories, we found highly similar proportions of genes residing within each of the three patterns (**Figures 4C, D**; N=1081 DE_D2_, N=100 DE_D14_, N=70 DE_D2, D14_).

Among the small percentage of retained and induced features, we explored to what extent peaks and genes following the same trajectories (i.e., retained peaks and retained genes; induced peaks and induced genes) were related to each other. Since there were more genes and peaks within the “induced” trajectory, we focused on this pattern first. We performed a gene ontology analysis on genes for which gene expression dynamics belonged to the “induced group” (**Figure 4E**) and compared it to enriched transcription factor motifs within induced H3K4me1 peaks - all induced peaks in **Figures 4A, B** and a broader set of peaks whose patterns followed a significant upward linear trajectory (**Figure 4F**). Among induced genes we found a strong enrichment of viral response pathways (e.g., “response to virus”; FDR = 7.27x10^-7^, **Figure 4E**) with 42% of these genes being direct targets of either the transcription factor Irf7 or Stat1 (for example, Oas1a) – both of which were also in the induced trajectory (**Figures 4G-I**). This suggests that the induced wave of novel gene expression as a whole may largely be driven by these transcription factors (**Figures S4C-G**). Among enriched motifs at increasing H3K4me1 peaks we found strong, significant enrichment of IRF/ISRE motifs (**Figure 4J**) which, on the other hand, were not enriched within the remainder of ‘not induced H3K4me1 peaks’ defined as peaks responsive to BG at one or more time points but not categorized as an increasing peak in **Figure 4F**. These motifs were enriched selectively among increasing H3K4me1 peaks, indicating the specificity of IRF motifs enrichments to the induced trajectory and demonstrating consistency with the finding that viral response pathways were only enriched among induced genes. When we assigned each peak to its closest gene, we found that induced or non-persistent peaks enriched significantly for induced or non-persistent genes, respectively (P_inc_ = 8.55x10^-5^, P_dec_ = 1.07x10^-42^; **Figure 4K**). Constant peaks enriched for constant genes with a higher but near-significant p-value (P_cons_ = 0.195) likely due to the low power arising from the small total number of genes and peaks within this trajectory.

Collectively our data demonstrate that H3K4me1 peaks that are significant at D14 represent peaks that followed either a retained or induced trajectory following BG washout and are significantly associated with transcription factor networks following the same expression patterns.

### 3.5 Changes in H3K4me1 and gene expression are associated with evolving iBMDM functional responses

**Figure 5:**
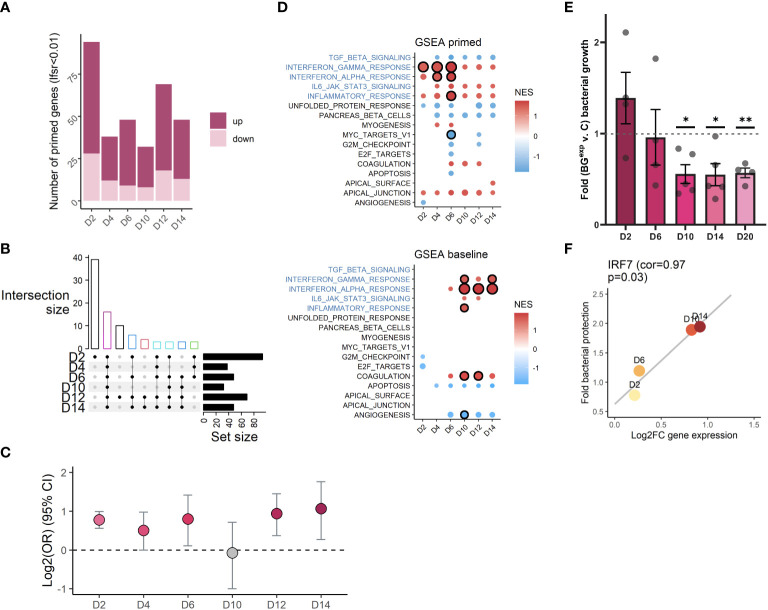
Changes in H3K4me1 and gene expression are associated with evolving iBMDM functional responses. **(A)** Bar plot quantification of the number of primed genes per time point (lfsr < 0.01). **(B)** Upset plot showing overlap of primed genes across timepoints (showing only combinations with n>=3 genes). Bar colored by number of shared time points (black=1, red=2, green=3, dark blue=4, teal = 5, magenta=6). **(C)** Log2 odds ratio (y axis) enrichment of primed genes at each time point among genes annotated to significant peaks at the same time point. **(D)** Significantly enriched (circle with black outline, FDR<0.1) hallmark immune pathways among primed genes (top panel) and differentially expressed genes at baseline (bottom panel). Pathways with a circle but no outline have a p-value < 0.05. Circle size is scaled to –log10(padj). Color is scaled to normalized enrichment score (NES). **(E)** Fold change bacterial growth comparing BG^exp^ relative to timepoint and experiment paired control (ratio paired t-test *p<=0.05, **p<=0.01). **(F)** Scatterplot showing log2FC expression of IRF7 at D2, D6, D10, and D14 on the x-axis and bacterial protection (inverse average fold change value at the respective timepoint shown in E) on the y-axis, with line of best fit (p=0.03, pearson correlation = 0.967).

It has been hypothesized that the presence of altered levels of H3K4me1 at enhancer regions may enable transcription factors to rapidly upregulate gene expression upon a secondary immune challenge, a phenomenon that is often referred to as “priming” (1, 2). For example, human monocytes stimulated with BG had a stronger proinflammatory cytokine response to a secondary lipopolysaccharide (LPS) or Pam3CSK challenge compared to naïve monocytes (3, 6, 7), and harbored altered histone PTMs at nearby sites. However, most studies have only focused on assessing priming at a single time point. Since our time course approach revealed that significant H3K4me1 signatures varied across time, we hypothesized that the repertoire of primed genes may differ depending on the timepoint interrogated. To assess responses of BG^exp^ cells to secondary challenges, we stimulated a subset of control and BG^exp^ cells with Pam3CSK4 for 5 hours at D2, D4, D6, D10, D12, and D14 and compared their ability to rapidly upregulate genes in response to the secondary stimulation. We detected 120 primed genes (lfsr<0.01) whose log2FC response to secondary stimulation in BG^exp^ cells was significantly different compared to controls for at least one time point (**Figure 5A** local false sign rate (lfsr) < 0.01; **Figures S5A-C**; **Table S7**). The largest total number of primed genes was detected at D2, also the time point with the greatest number of differences in all histone modifications profiled. We found various levels of overlap between primed genes at different time points (**Figure 5B**). The largest percentage of primed genes were specific to the first time point, D2, while the second most common pattern was shared priming across all time points. We also observed genes which were primed only at later timepoints (D12, D14, or both) and genes whose direction of priming reversed (**Figures S5D-F**), consistent with the idea that the set of primed genes identified at one time point could differ significantly from the primed genes at another time point, arguably because of the lack of overlap between significant H3K4me1 peaks at D2 compared to D14. In further support of this point, we found that when significant H3K4me1 peaks at each timepoint were assigned to their closest genes, time-matched primed genes were enriched at every time point except D10 (**Figure 5C**) demonstrating a significant link between the unique repertoire of H3K4me1 signatures and primed genes at each time point.

Interestingly, GSEA on primed genes revealed that the strongest priming occurred at interferon alpha and gamma pathways between D2 and D6 – also the pathways whose baseline expression was induced most strongly at later time points beginning at D10 (**Figure 5D**). We speculate a model in which the reinduced expression of interferon and inflammatory pathways may be related to their priming at early timepoints. Given that priming at D2 and D4 still intersects with a state of continued NFκB activity and differential expression of other genes, it is not unfathomable that cells could self-engage their own primed pathways post-washout, using residually active transcription factors which could bind to primed regions to drive the new differential gene expression waves emerging at later timepoints. This would explain why the pathways most strongly responsive to BG stimulation, the most strongly primed pathways, and the pathways re-engaged post-washout are so strongly overlapping (**Figures 3F**, **5D**).

Finally, we tested whether timepoint specific differential gene expression, epigenetics, and/or priming, may impact functional responses of iBMDMs to bacterial infection, mimicking a more biologically relevant situation. We infected control and BG^exp^ iBMDMs with *S. Typhimurium* at D2, D6, D10, D14, and D20 after BG washout and quantified the ability of iBMDMs to control bacterial growth over a 4-hour period using a CFU assay (**Figure 5E**). In these experiments BG^exp^ cells gained an approximately 2-fold increase in the ability to control bacterial growth, however this “trained” phenotype emerged only at later timepoints (D10-D20; **Figure 5E**) and followed kinetics highly correlated with the induced wave of baseline interferon expression we previously observed (**Figure 5F**). Thus, functional protection was much more closely correlated to the newly emerging gene expression program, H3K4me1 marks, and primed genes found at later timepoints, compared to the retained signatures of activation still present at D2. These data point to a role for newly induced changes post-washout, rather than the immediate post-BG priming, in shaping functional outcomes in response to secondary pathogen encounters.

## 4 Discussion

In this study, we performed time course ChIP- and RNA-sequencing to track the gene expression and epigenetic dynamics of dividing macrophages following a 24-hour stimulation with BG. Our results demonstrate that a completely isolated, dividing macrophage population can harbor long-term changes in levels of H3K4me1 for at least 14 cell divisions following BG stimulation. Notably, these differences made up a very small proportion of total stimulus responsive H3K4me1 peaks, of which over 85% were lost with kinetics matching a model of passive decay, in accordance with the notion that dividing cells lack the ability to actively copy PTMs during DNA replication (13–15). Nonetheless, the residual H3K4me1 “memory” was present even after all NFκB activity had returned to baseline levels, demonstrating that H3K4me1 signatures can be detected independently of acute cell activation. In contrast, the other two histone modifications profiled – H3K4me3 and H3K27Ac – returned to baseline levels after a few cell divisions post-BG stimulation, demonstrating a potentially unique role for H3K4me1 as a marker of previous BG exposure. These data are consistent with previous findings that trained immunity can induce epigenetic reprogramming of long-lived cell populations (18, 21–23), particularly at enhancer regions - which are the primary sites of H3K4me1.

A central question in the field of trained immunity is how to reconcile the observation that stimulus-induced epigenetic alterations can be observed over many cell divisions – in our system for at least 14 – and the fact that histone PTMs cannot be actively copied. We found that BG^exp^ cells evolve new sets of DE genes and associated H3K4me1 peaks during the washout phase, which largely contributed to why we were able to detect significant H3K4me1 peaks, even at D14. We detected a novel set of interferon genes and transcription factors that were induced during the washout period and that were accompanied by increased H3K4me1 levels at peaks containing IRF/ISRE motifs. We found overall patterns of differential gene expression and H3K4me1 to be strongly correlated, supporting our hypothesis that differential transcription factor activity may be driving, and required for, the continued presence of H3K4me1 differences. These results suggest that “memory” may be a misleading term, at least in our system. Rather, the gene expression and epigenetic programs continue to be shaped, even after the stimulus has been removed, and the cells are no longer chronically activated. IRF ChIP-seq analyses aimed at directly determining the binding patterns of IRF transcription factors, will help to further reinforce the link between IRFs and the transcriptional and epigenetic programs induced in response to BG.

Fundamentally, our model suggests that a fine-tuned series of events with specific timing is essential to induce the phenotypes observed here. Cells must be primed at an early timepoint during which they still remain marginally active, allowing transcription factors with remaining activity to engage the primed pathways on their own, thus driving the subsequent waves of gene expression and epigenetic changes detected at later timepoints. This model suggests that cells which become deactivated too quickly, or cells which don’t become primed during the time frame of this continued activation will likely not induce new gene expression programs or H3K4me1 signatures. The fact that a kind of “perfect storm” of co-occurring features may be required to induce long-lasting epigenetic signatures is ultimately consistent with the fact that trained immunity has proven to be incredibly sensitive to experimental design. We propose that the central mechanistic findings in this study – passive histone decay, the coupling of transcription factor activity with induced or retained H3K4me1 signatures, timepoint specific priming and epigenetic signatures, and the ability of early priming to drive stimulus-experienced cells down this path of continuous change – may represent general requirements for trained immunity within dividing cell populations devoid of any help from other cell populations of a pro-inflammatory microenvironment. Ultimately, it remains unclear whether the long-lasting changes in H3K4me1 observed in our data are required for the functional reprograming of macrophages or simply just correlate with variation in the activity of specific TFs (in our specific setting primarily IRFs). We hope that our findings will motivate future studies focused on epigenetic editing of macrophages to dissect whether particular epigenetic changes alone are necessary and sufficient for the induction of innate immune memory or, if instead, the cornerstone of innate memory is the long-term rewiring of baseline TF activity in response to an initial challenge.

## Data availability statement

The data presented in the study are deposited in the GEO repository, accession number GSE225855.

## Author contributions

LB conceived the project and directed the study. SS and LB designed the experiments. SS performed the experiments. SS led the computational analyses with contributions from RA-G. SS and LB wrote the manuscript, with input from all authors. All authors contributed to the article and approved the submitted version.
