# Supplementary material for: Transmission of stimulus-induced epigenetic changes through cell division is coupled to continuous transcription factor activity
Source: Front Immunol. 2023 Mar 14;14:1129577. doi: 10.3389/fimmu.2023.1129577 (PMC10043173; doi:10.3389/fimmu.2023.1129577)
Supplement: Supplementary file 1 [file DataSheet_1.pdf]

Supplementary Material

Transmission of stimulus-induced epigenetic changes through cell division is coupled to continuous transcription factor activity

Sarah Sun[1,2], Raúl Aguirre-Gamboa[3,4], Luis B. Barreiro[1,3,4]*

*** Correspondence: Barreiro, Luis B: lbarreiro@uchicago.edu**

1 Supplementary Data

This PDF file includes:

Figures S1 to S5

Table Legends S1 to S7

Data Sharing: All sequence data has been deposited in GEO (GSE225855). Any additional information required to reanalyze the data reported in this paper is available from the lead contact upon request.

2 Supplementary Figures and Tables

2.1 Supplementary Figures

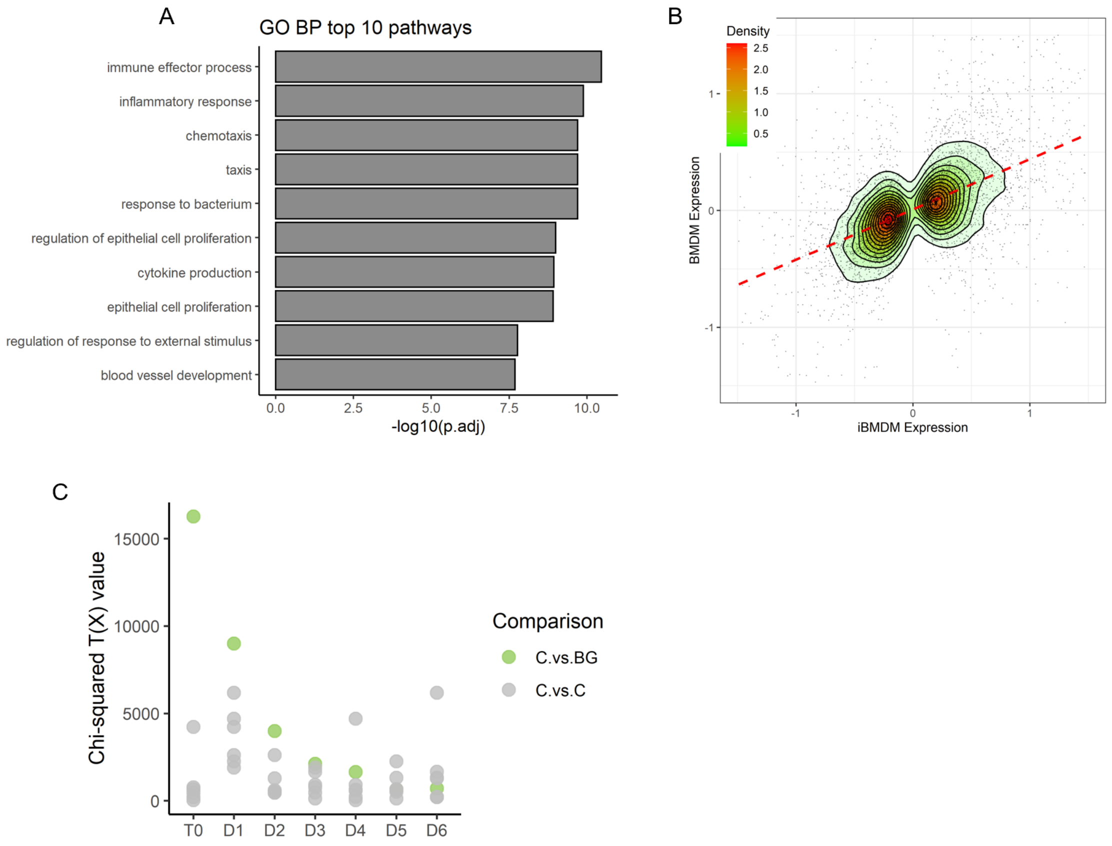

Supplementary Figure 1. Effect of BG on iBMDM$^{NF\kappa B\text{-}GFP}$. (A) Gene ontology analysis with Biological process (BP) pathways performed on significant genes (FDR<0.05, abs(logFC)>1 after 7 hrs beta glucan stimulation). The top 10 pathways with the lowest p.adj values are shown. (B) Correlation analysis of DE genes in primary BMDMs and iBMDMs at 7 hours of BG stimulation (Pearson's r = 0.61, P< 1x10-16, 82% concordant in the direction of the effects, red dotted line indicates line of best fit). (C) Statistical analysis comparing GFP levels from control cells and BG experienced cells after every cell division post washout. At every time point a Chi-squared T(x) statistic was computed to compare control GFP levels with GFP levels in the timepoint paired BG sample (C.vs.BG) and with all other controls collected at other time points (C.vs.C) to establish a significance cutoff.

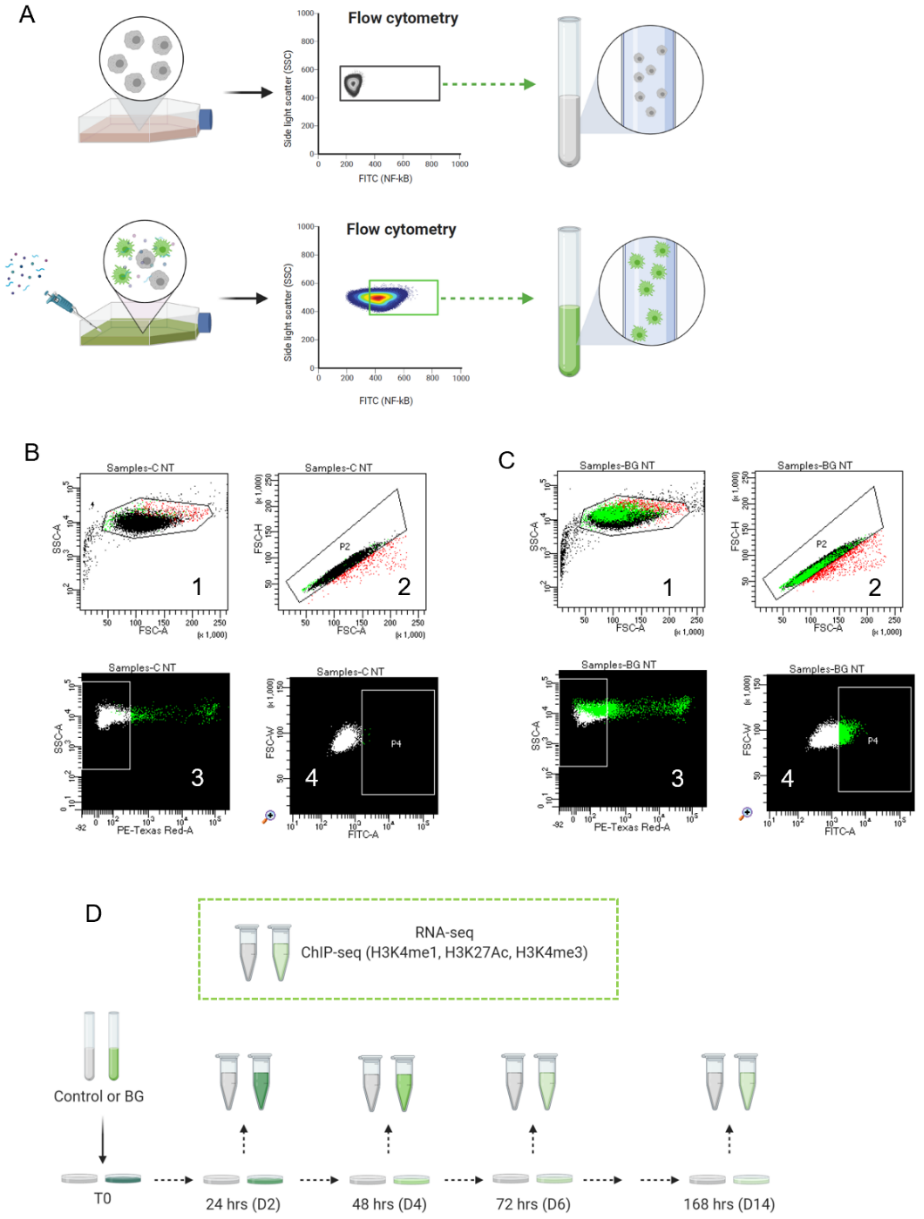

Supplementary Figure 2. Time course experiment design. (A) Cell stimulation and sorting workflow. iBMDM$^{NF\kappa B\text{-}GFP}$ cells are left unstimulated (C, top) or stimulated with 30 µg/mL β-glucan (BG, bottom) for 24 hours. After 24 hours C and BG cells were collected and sorted (C: gated on live, single cells; BG: gated on live, single, GFP+ cells). (B) Example gating, control sample (all PE-Texas Red negative cells in gate 3 were sorted). (C) Example gating after 24 hrs stimulation with 30 ug/mL beta glucan (all PE-Texas Red negative, GFP+ cells in gate 4 were sorted). (D) Sorted C and BG^{GFP+} cells are returned to cell culture. Every two days, 600,000 C and BG^{exp} cells were passaged into new flasks to prevent overcrowding. Aliquots of C and BG^{exp} cells were collected every 24 hours up to 168 hours (D2-D14) and used for downstream RNA-seq and ChIP-seq analysis.

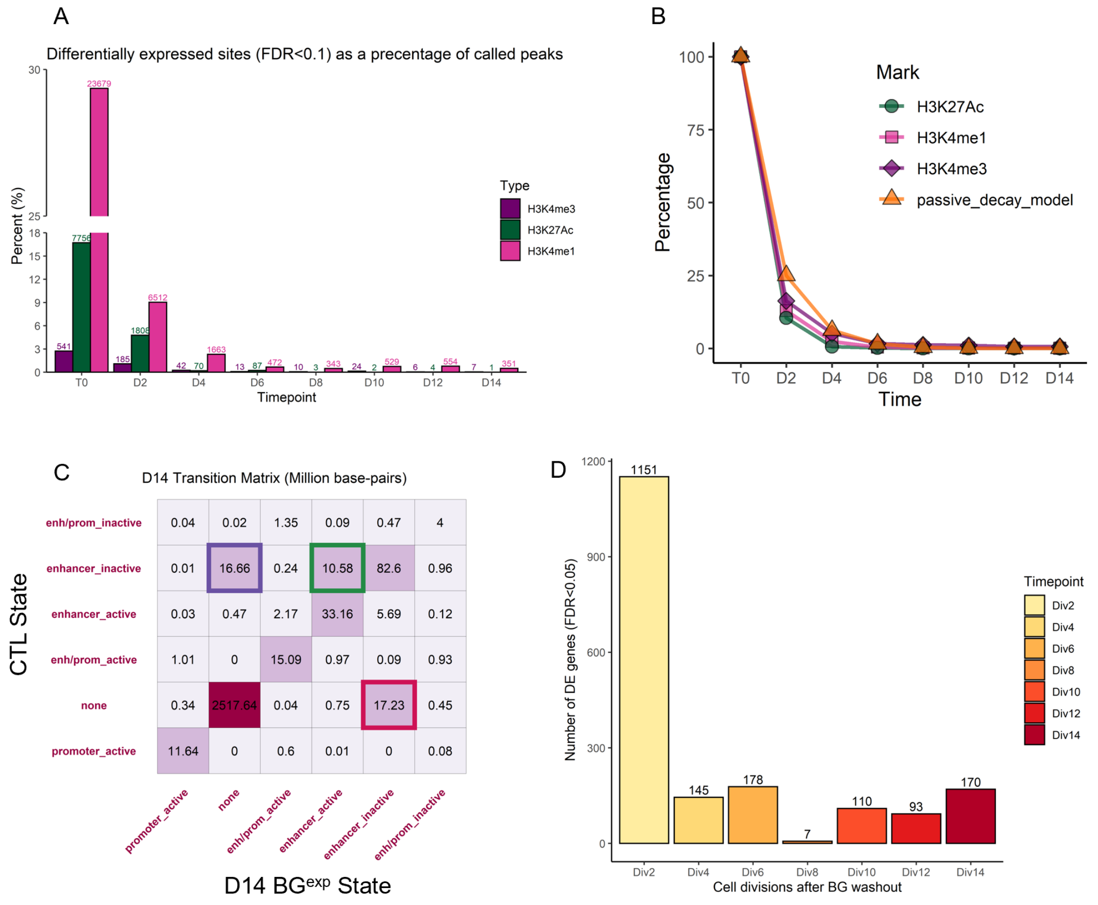

Supplementary Figure 3. Impact of BG on H3K4me1, H3K27Ac, H3K4me3, and gene expression post washout. (A) Bar-plot showing the number of sites with differential abundance of H3K4me1, H3K4me3, and H3K27Ac (FDR<0.1) at each time point determined by ChIP-sequencing (data representative of 2-3 C and BG^{exp} replicates per time point). Bar height indicates the number of significant peaks as a percentage of all called peaks for each particular histone modification. The number above the bar indicates the total number of significant peaks. (B) Line plot showing the percentage of significant peaks at T0 (FDR<0.05) remaining significant at each subsequent timepoint. Orange line models expected percentages assuming 50% loss with each cell division. (C) Transition matrix displaying the proportion of base pairs in each transition state at D14. ChromHMM was used to segment the genome into 6 states using H3K4me1, H3K4me3, and H3K27Ac ChIP-seq data. Separate segmentations were performed with control and BG D14 ChIP-seq profiles. State assignments between control and BG D14 segmentation outputs were compared across the entire genome using 200 base pairs as the minimal unit. (D) Bar-plot showing the number of differentially expressed genes (FDR<0.05) at each time point determined by RNA-sequencing (data representative of 3 C and BG^{exp} replicates per time point)

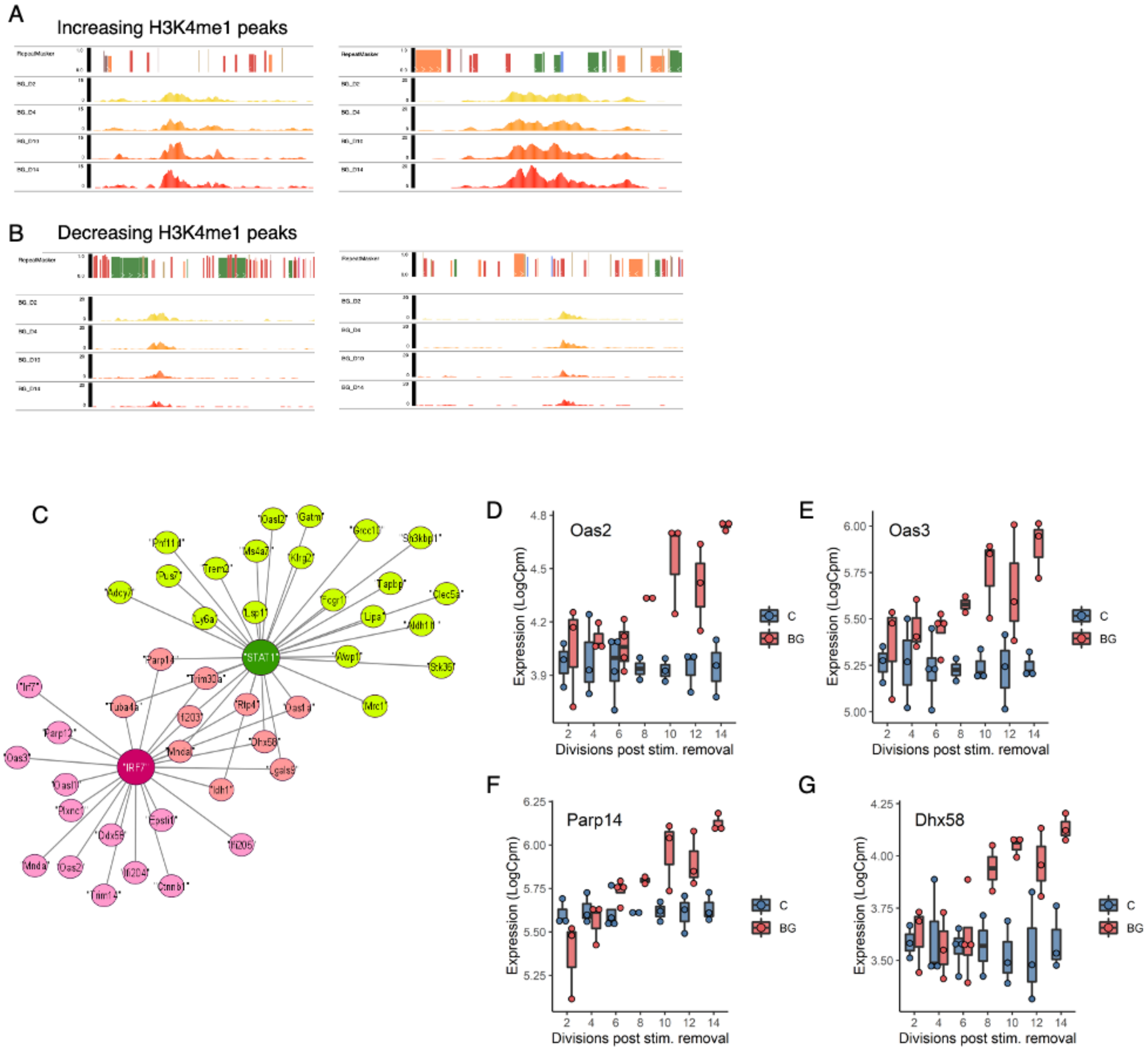

Supplementary Figure 4. Induced genes post washout. (A) Genome browser snapshot of increasing H3K4me1 peaks (*left* – Peak#6623, *right* – Peak#89198). (B) Genome browser snapshot of decreasing H3K4me1 peaks (*left* – Peak#45842, *right* – Peak#61605). (C) Network showing all genes in the induced trajectory whose promoter contains a Stat1 or Irf7 motif in the interferome database. (D-G) Examples of induced genes

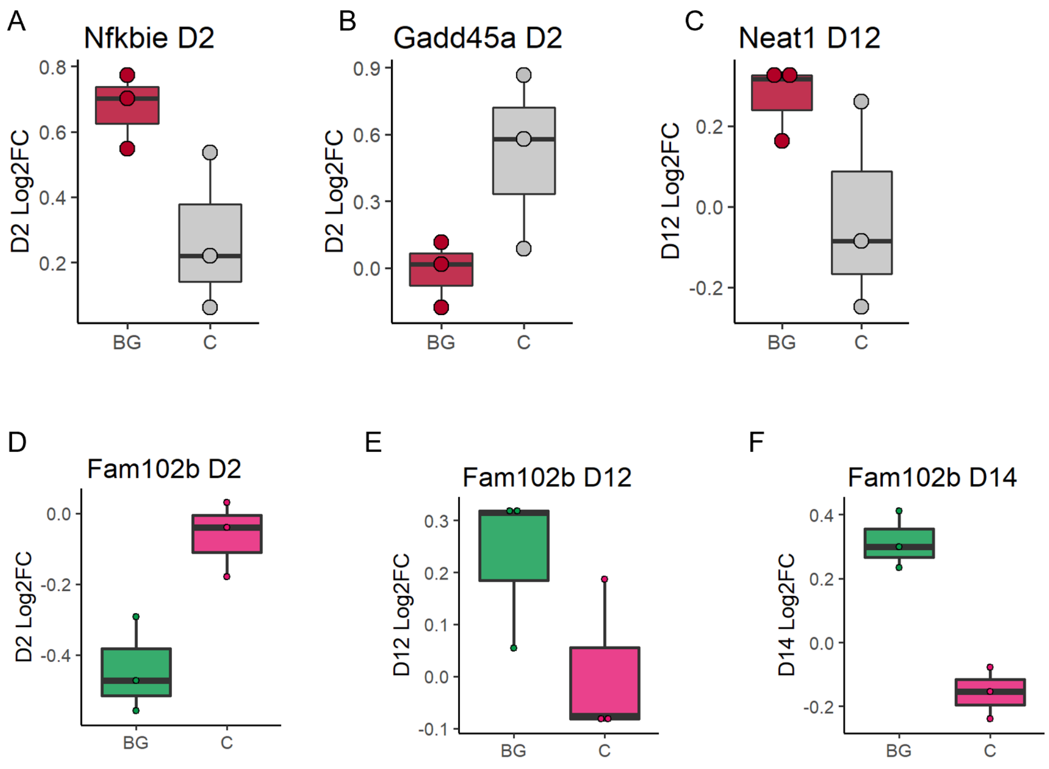

Supplementary Figure 5. BG-stimulation induces time point specific priming. (A-C) Example boxplots for primed genes, (A) Nfkbie D2, (B) Gadd45a D2, (C) Neat1 D12. (D-F) Example of a gene whose direction of priming reverses. Boxplots show log2FC Fam102b expression after 5 hours Pam stimulation at D2 (D), D12 (E), and D14 (F)

3 Supplementary Tables captions

Table S1. Summary statistics for differential gene expression analysis performed on RNA-seq samples at each timepoint during beta glucan stimulation, related to figure 1

Table S2. Summary statistics (top 30,000 peaks ranked by P-value) for differential abundance analysis performed on H3K4me1 ChIP-seq samples at each timepoint following beta glucan washout, related to figure 2

Table S3. Summary statistics (top 1000 peaks ranked by P-value) for differential abundance analysis performed on H3K4me3 ChIP-seq samples at each timepoint following beta glucan washout, related to figure 2

Table S4. Summary statistics (top 8000 peaks ranked by P-value) for differential abundance analysis performed on H3K27Ac ChIP-seq samples at each timepoint following beta glucan washout, related to figure 2

Table S5. Chromosome and coordinate location for each called peak from H3K4me1, H3K4me3, and H3K27Ac ChIP-sequencing, related to figure 2

Table S6. Summary statistics for differential gene expression analysis performed on RNA-seq samples at each timepoint following beta glucan washout, related to figure 3

Table S7. LFSR and posterior means output for each gene at each timepoint from primed genes analysis, related to figure 5
